# Association between increased expression of endothelial isoform of nitric oxide synthase in the human fallopian tube and tubal ectopic pregnancy

**Published:** 2014-01

**Authors:** Leyla Fath Bayati, Marefat Ghaffari Novin, Fatemeh Fadaei Fathabadi, Abbas Piryaei, Mohammad Hasan Heidari, Mozhgan Bandehpour, Mohsen Norouzian, Mahdi Alizadeh Parhizgar, Mahmood Shakooriyan Fard

**Affiliations:** 1 *Department of Biology and Anatomical Sciences, Faculty of Medicine, Shahid Beheshti University of Medical Sciences, Tehran, Iran.*; 2 *Cellular and Molecular Biology Research Center, Shahid Beheshti University of Medical Sciences, Tehran, Iran.*; 3 *Department of Pathology, Kamkar Arab-Niya Hospital, Qom University of Medical Sciences, Qom, Iran.*

**Keywords:** *Ectopic pregnancy*, *Nitric oxide*, *Immunohistochemistry*, *Fallopian tube*

## Abstract

**Background:** Tubal ectopic pregnancy (tEP) is the most common type of extra-uterine pregnancy and the most common cause of maternal mortality. Nitric oxide (NO) is a molecule that incorporates in many physiological processes of female reproductive system. Recent studies have demonstrated the possible role of endothelial isoform of nitric oxide synthase (eNOS) enzyme in the regulation of many reproductive events that occur in the fallopian tube (FT).

**Objective: **The aim of this study was to evaluate the expression of eNOS in the FTs of women with tEP.

**Materials and Methods:** In this case-control study, a total number of 30FTs samples were obtained from three groups including: 10 FTs of women that bearing an EP, 10 FTs from the non-pregnant women at luteal phase of the menstrual cycle, and 10 FTs of healthy pregnant women (n=10). Samples were fixed in 10% buffered formalin and then were evaluated by immunohistochemistry.

**Results:** Localization of eNOS was seen in secretory and ciliated luminal epithelium and vascular endothelium of all groups. However, we did not observed the expression of eNOS in smooth muscle cells of all groups. Expression of eNOS in luminal epithelium of women with EP compared to non-pregnant women at luteal phase of menstrual cycle and healthy pregnant group showed statistically significant increase (p=0.00). Significant difference in expression of eNOS was not observed in luminal epithelium of FTs of women at luteal phase compared to healthy pregnant groups (p=0.78).

**Conclusion:** This study indicates that changes in expression of eNOS in luminal epithelium of FT may lead to development of EP.

This article extracted from M.Sc. thesis. (Leyla Fath Bayati)

## Introduction

Ectopic pregnancy (EP), the implantation of a fertilized ovum outside the endometrial lining of uterine cavity, accounts for leading cause of morbidity and mortality in women reproductive-age (1-4). It accounts for 1.5-2% of all pregnancies in the western world and more than 95% of EP occurs in the fallopian tubes (FTs), mainly in the ampulla region (5, 6). The etiological causes for EP include damage to the fallopian tube due to previous tubal surgery, pelvic inflammatory disease, cigarette smoking, assisted reproductive technology, endometriosis and history of EP (1, 7-11). Maintaining of embryo within the fallopian tube as a result of improper transport and deleterious tubal alteration can lead to tubal ectopic pregnancy (tEP) (12). Successful tubal transport of gametes and blastocyst is substantial for fertilization, early embryo development, and intrauterine pregnancy. Effective transportation within the tube is obtained by interactions between muscle contractions; ciliary beating and factors that secreted by fallopian tube (13).

Ovarian hormones and other factors produced by the fallopian tube, such as prostaglandins, Nitric oxide (NO), prostacyclin and cAMP may also regulate muscle contraction and play significant role in embryo transport (14-21). Also, transport of embryo within the fallopian tube is influenced by ciliary beats which is regulated by factors such as sex steroid hormones and IL-6 (22-24). Results of the previous studies demonstrated that ovarian follicular fluid contains factors that could affect ciliary beating in the fallopian tube during ovulation period (25).

Findings of previous study shown that NO could affect the contraction of fallopian tube smooth muscle and ciliary beat frequency in epithelial cells of airway via cyclic guanosine mono phosphate (cGMP) pathway (17, 18, 26, 27). Others reported that endothelin, a potent contracting factor are produced by bovine oviduct and is able to induce contraction of oviduct (28-30). They also demonstrated that the contractile properties of endothelin-1 on bovine oviducts are enhanced by the presence of N-monomethyl-L-arginine mono acetate (L-NMMA), an inhibitor of NO synthesis. These findings provided the first indirect evidence for endogenous synthesis of NO within the oviduct (30).

NO is a free radical that is produced from L-arginine in the process of its conversion to L-citrullin by the action of nitric oxide synthase (NOS) enzyme, an enzyme existing in three isoforms (31). Neuronal NOS (nNOS or NOS1) and endothelial NOS (eNOS or NOS3), also referred to as constitutive NOS (cNOS), are responsible for the continuous basal release of NO and both require calcium/ calmodulin for activation (32, 33). Third isoform of NOS is an inducible calcium-independent form (iNOS or NOS2) that is expressed only in response to inflammatory cytokines and lipopolysaccharides (LPS) (34, 35). The three isoforms of NOS are products of separate genes that share 50-60% amino acid homology (36).

NO has different physiological activity, including regulation of vascular resistance, contribution in cellular injury, and signal transduction (37-39). NO is a double-edged sword. At low concentration, it alters intracellular Ca^2+^ levels and induces smooth muscle relaxation (40). But at high concentration, it can lead to immune reaction and tissue damage (41). NO has an important role in smooth muscle relaxation and ciliary activity so has attracted many researches focusing on these fields (38, 23). In fallopian tube impaired ciliary beating and smooth muscle cells contractions may lead to infertility or tubal implantation of blastocyst and the development of EP (13). As mentioned above, production of NO by the human fallopian tube has been previously reported, and effect of this messenger molecule on relaxation of tubal smooth muscle cells was documented (17, 42, 43). Also, in a recent study, iNOS mRNA and protein levels were shown to be greater in the fallopian tube of women with tEP compared with pseudo pregnant women (44).

In the present study, we investigated the expression of eNOS in different group, because eNOS is the main isoform of NOS expressed in the oviduct (27). Our study investigated that whether the fallopian tubes bearing an EP differed from normal tubal tissues in expression of eNOS. As mentioned in previous studies human Fallopian tube is the most frequent site for EP. We hypothesized that regulation of eNOS may be altered in the process of EP, and this can be considered as one of potential causes of inability to successful pregnancy.

## Materials and methods

All techniques, methods and selection of patients were approved by ethical committee of Shahid Beheshti University of Medical Sciences. Tissue was obtained from patients at Vali-e-asr and Izadi Hospitals in Qom, Iran. The patients were informed in detail about the study and written consent was obtained from each case. Time of sample collection lasted from August 2011 to March 2012.

**Sample collection and preparation of sections**

In this case control study, immune histochemical localization of eNOS were assessed in FTs of women in three groups including, FTs of women bearing an EP (n=10), FTs of women in luteal phase of menstrual cycle (n=10) and FTs of healthy pregnant women (n=10).

**Case Group**

The ectopic pregnancy group (n=10) consisted of women (29.00±3.70 years, range 24-35 and average estimated gestational age calculated from the last menstrual period (LMP) was 5.66±2.55 weeks: data shown at mean±SE) that bearing an ectopic pregnancy. Salpingectomy in this group was performed on clinical management ground. All participants conceived spontaneously and were not taking exogenous progesterone. We excluded cases in which the EP was due to reflux after in vitro fertilization (IVF). Also we excluded the women who were cigarette smoker or have prior history of pelvic inflammatory disease (PID).

**Control Group**

The luteal phase group (n=10) consisted of women that were at luteal phase of menstrual cycle (38.33 ± 6.63 years, range 28-49 years) who presented for total abdominal hysterectomy (TAH) due to benign disease that not affected the fallopian tubes. All women at luteal phase group who donated FTs had regular menstrual cycles and we excluded the women that had evidence of active infection or tubal disorders (44). 

The healthy pregnant group (n=10) consisted of women (35.16±4.04 years, range 28-40 years) that were undergoing elective caesarean section (CS) at the time of labour. For this group tubal ligation was performed during CS because they will intend to prevent of pregnancy in the future. So, we collected the FTs samples of these women without any problems and according to their written consent. If women had a tubal disorders, or evidence of active infection, or following induction of labor, they were excluded from the study. We chose healthy pregnant women at term (>37 weeks’ gestation) and non-pregnant women at luteal phase of menstrual cycle as a control group. 

Women who bore an EP were chosen as a case group. In this study, the ampullary region of the excised tubes from the three groups was identified and a small section was cut from each region. For the case group, the FTs were excised at least 1cm away from the implantation site to avoid collecting any embryonic or trophoblastic tissue (44). Then samples were washed in 0.1 mol/L phosphate-buffered saline (PBS) three times and were immediately fixed in 10% buffered formalin for immunohistochemistry evaluation.

**Immunohistochemistry**

After fixation, tissues were embedded in paraffin and later cut into 3 μm sections and placed on poly-L Lysine coated slides. Immuno histochemical protocol (horse-radish-peroxidase) was used to visualize the intensity and distribution of eNOS immune staining between three groups. A polyclonal rabbit antihuman antibody was used to detect eNOS (ABcam Company, UK). Paraffin-embedded tubal tissue sections (3 μm) were deparaffinized in xylene and rehydrated in ethanol series (100%, 96%, and 80%). Antigen retrieval was performed in a microwave in 10 mmol/L citric acid buffer, pH 6.0, for 14 minutes. 

Slides were allowed to be cool at room temperature and were washed three times in PBS before being incubated with methanol-H_2_O_2_ (96:4, v/v) for 15 minutes to inhibit endogenous peroxidase. The slides were then exposed to1.5% normal gaot serum (DAKO Co, Denmark) in humidified chambers for 30 minutes at room temperature to avoid unspecific bindings of antibody. 

The primary antibody against eNOS (1:100) was placed on slides and incubated at 37^o^C for 1h. After rinsing for several times with buffer (0.1 M of phosphate-buffered saline with a pH of 7.4), the sections were incubated with the secondary antibody, a goat anti rabbit IgG (AB cam Co., UK) diluted 1:1000 in PBS. Incubation with the secondary antibody was performed for 1h at 37^o^C in incubator. Then, 3,3-diaminobenzidine in H_2_O_2_ (DAKO Co., Denmark) was added to the sections for 15 minutes. Thereafter, the sections were counterstained with hematoxylin and mounted. To validate the specificity of the immune staining, the positive and negative controls were carried out. We used human full term placenta tissue as external positive control (38, 39, 45). 

For negative controls, slides were incubated similarly to above protocol, but phosphate-buffered saline replaced the primary antibody. Immuno histochemical staining was evaluated by two independent persons using a light microscope at ×40 and ×400 magnifications. The persons were blinded to the source of tissue, and they agreed on the intensity of staining according to the following semi quantitative scale that reported in the previous studies (43, 44). 0= negative; 1= equivocally positive; 2-4= weakly positive; 5-7= positive; 8-10= strongly positive. The average of these observations was recorded as the final results of study. The results were expressed as percentage optical density (39).

**Statistical analysis**

Data was processed by SPSS 14 Software (SPSS/PC- 14). The data were expressed as Mean±SE which was used for ANOVA analysis and Tukey post Hoc test. The p<0.05 was considered statistically significant

## Results

**Immunohistochemistry of eNOS**

The demographics of the three groups of women studied are shown in Table I. The mean age of EP group was 29.00±3.70 years that was lower than other groups (Luteal phase group with mean age of 38.33±6.63 and healthy pregnant group with mean age of 35.16±4.04). Because previous studies did not demonstrate the age of women as the risk factors of EP, we didn’t estimate the age as potential effector on the results of this study (1-6, 8). 

**Figure 1:**
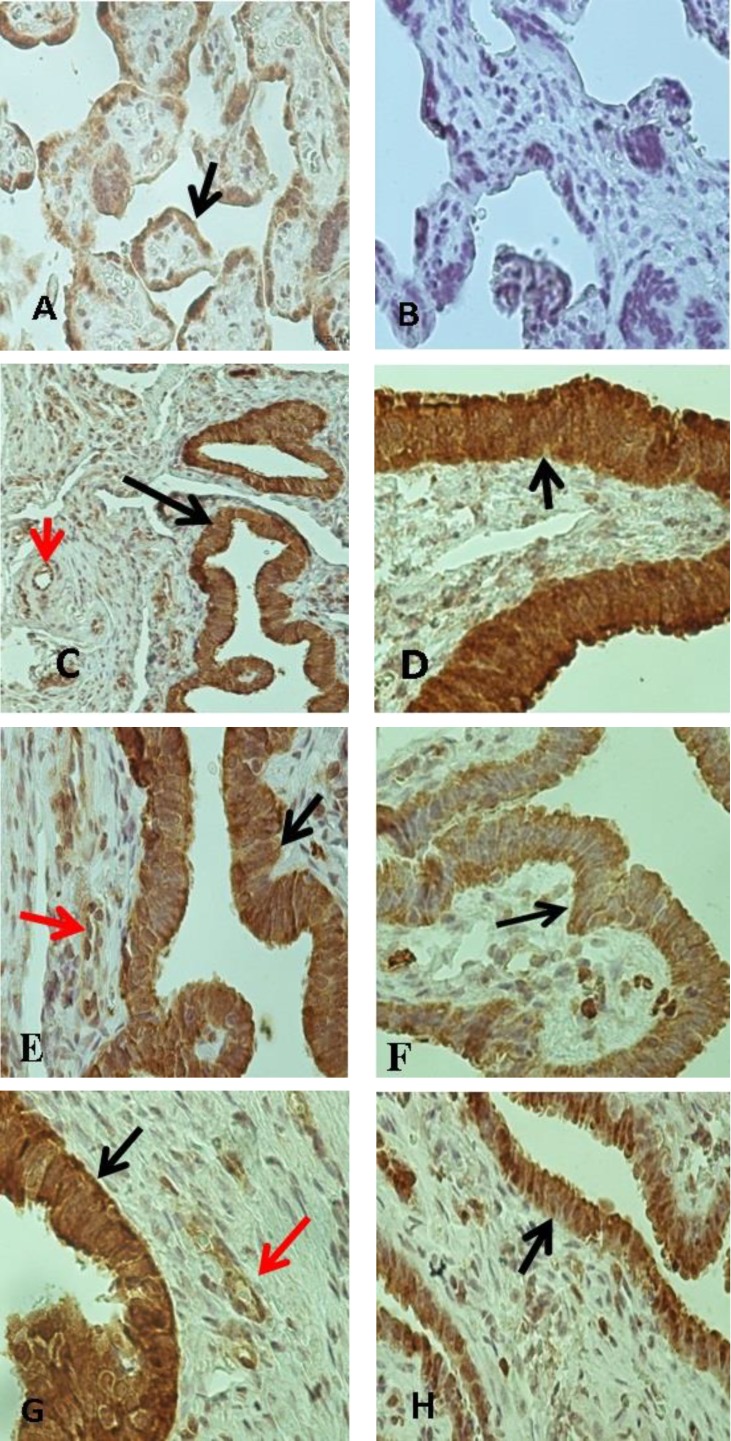
(A) Immunohistochemical localization of eNOS enzyme in Human placenta that was used as a positive control. (B) shows staining of placenta tissue without using primary antibody (negative control). Immunohistochemical localization of eNOS enzyme in the ampullary regions of human fallopian tube sections collected from ectopic pregnancy (C and D), luteal phase group (E and F) and healthy pregnant group (G and H). Blank arrows denote the strong immunostaining in the luminal epithelium and red arrows denote to endothelial lining of blood vessels. (A and B were taken at ×10 magnification. D, E, F and G were taken at ×40 magnification. C and H taken at ×10 magnification

Women at luteal phase and healthy pregnant groups decided not to have childbearing in the future, this is naturally that why the EP group had mean age lower than other groups. A positive control for eNOS staining consisted of human placenta in which eNOS was consistently detected (Figure 1A). Placenta tissues that incubated in PBS in the absence of primary antibody (negative control) showed no staining for eNOS antibody (Figure 1B). 

Positive eNOS antibody staining was observed in all tissue samples from three groups, including EP (Figure 1 C, 1D), luteal phase (Figure 1E, 1F) and healthy pregnant groups (Figure 1G, 1H). Our studies showed that (in three groups) special cells such as ciliated and secretory cells of luminal epithelium and endothelial lining of blood vessels had positive staining for antibody to eNOS. eNOS antibody strongly labeled the entire part of the epithelial cells. Also, our data showed that endothelial wall of blood vessels had positive staining for eNOS in all groups (Table II, Figure1). We did not observe any differences in expression of eNOS in endothelium of blood vessels between three groups. 

**Figure 2:**
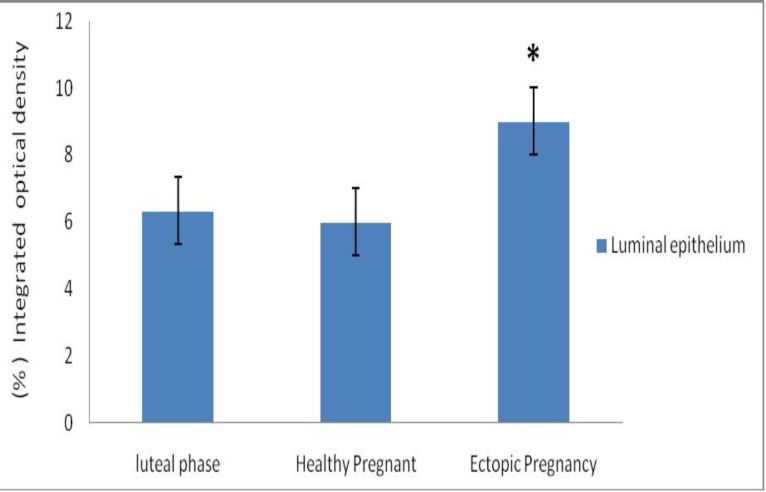
eNOS protein expression by image analysis in luminal epithelium areas of fallopian tubes in ectopic pregnancy group compared with healthy pregnant and luteal phase groups. Data were analyzed by one-way ANOVA and *p<0.05.

No staining was detected in the smooth muscle cells of FT sections from all of groups. All of the epithelial cells were stained in three groups. Although, expression of eNOS in luminal epithelium of FT samples from EP group was higher in comparison with luteal phase and healthy pregnant groups. According to these data, differences between EP group and luteal phase group (p=0.00) and healthy pregnant group (p=0.00) was statistically significant (Figure 2). Significant differences were not seen in immuno staining between the luteal phase and healthy pregnant groups (p=0.78) in expression of eNOS (Figure 2).

**Table I:** Demographic characteristic of the study population

**Groups**	**Ectopic Pregnancy**	**Luteal phase**	**Healthy pregnant**
Age (years)	29.00 ± 3.70	38.33 ± 6.63	35.16 ± 4.04
Weight	60.50 ± 4.44	62.11 ± 4.78	59.08 ± 5.83
eNOS immunostaining	9.00 ± 0.53	6.33 ± .50	6.00 ± 1.65

Data are shown in mean±SE.

**Table II:** Intensity of immune-histochemical localization of eNOS protein in the FTs of three groups of women

**Groups**	**Endothelial Lining**	**Luminal Epithelium**
Healthy pregnant	6.00 ± 1.31	6.00 ± 1.62
Luteal phase	6.00 ± 1.12	6.33 ± 0.50
Ectopic pregnancy	6.00 ± 1.84	9.00 ± 0.53

Data are shown in mean±SE

**Table III:** Multiple Comparisons between groups in immunostaining of eNOS in luminal epithelium (Tukey HSD)

**(I) group**	**(J) group**	**Std. Error**	**Sig.** *	**95% Confidence Interval**	
				**Lower Bound**	**Upper Bound**
EP	Luteal phase	0.55566	0.000	1.2859	4.0474
	Healthy pregnant	0.52195	0.000	1.7030	4.2970

Psignificant differences (p<0.05) between EP/ Luteal phase group.

Psignificant differences (p<0.05) between EP/ Healthy pregnant group.

*. The mean difference is significant at the 0.05 level.

Multiple Comparisons between groups in immunostaining of eNOS (Tukey HSD):

P (EP/ Luteal phase)= 0.00

P (EP/ Healthy pregnant)= 0.00

P (luteal phase/ Healthy pregnant)= 0.78

P significant differences (p<0.05) between EP/ Luteal phase group

P significant differences (p<0.05) between EP/ Healthy pregnant group

## Discussion

Nitric oxide as a paracrine molecule regulates many physiological processes that occur in female reproductive system including: hormonal secretion, cervical repening, uterine relaxation and oviduct motility (17, 46). Because these functions are linked to the biology and physiology of various reproductive processes, several studies focused on the mechanism of NO regulation in the different organs of the reproductive system (47, 48). The present work was designed to investigate the existence and distribution of endothelial isoform of NOS in the ampullary sections from FTs samples of different groups of women. Results of this study showed that eNOS express in human FTs tissue of three groups: women with EP, non-pregnant women at luteal phase of menstrual cycle and healthy pregnant women at term. This finding is in accordance with previous studies of eNOS expression in human FT (42, 49). 

In this study, changes in expression of eNOS were found in the ciliated and secretory cells of luminal epithelium, where the most changes were in ectopic pregnancy group compared with luteal phase and healthy pregnant groups. This is the first study that shows an increase in the eNOS expression within the human fallopian tube bearing an ectopic pregnancy. We were interested in endothelial isoform of NOS because this isoform of NOS plays an important role in signal transduction associated with ciliary functions (27). 

Also, findings of previous studies have shown that eNOS is the main isoform of NOS expressed in the animal’s oviduct. So, they suggested that NO plays an important role in the regulation of oviduct functions (50). NO produced by NOS activity is essential factor in the regulation of smooth muscle cell tone, platelet aggregation, cell growth, apoptosis, neurotransmission and infection-induced immune reactions (51). NO is involved in normal pre-implantation embryo development but uncontrolled synthesis of this molecule could impair the development of embryo (52-54). The significant of NO has been linked to essential fallopian tube functions. The first evidence for the presence, as well as a physiological role of NO in regulating of oviduct contraction, was demonstrated in the bovine oviduct (30). 

Results of the previous studies demonstrated that administration of NO donors could decrease the contractility of the human fallopian tube (42). These findings emphasizes on the critical role of this signaling molecule on the human fallopian tube function. In addition, administration of NO inhibitors such as N-nitro-L-arginine methyl ester (L-NAME) causes to increase the contraction strength of smooth muscle of oviduct (17, 30). 

Also, other studies showed that estrogen and progesterone could regulate the production of NO and these sex steroid hormones increase synthesis of NO (46, 55). It has been shown that NO induces relaxation of fallopian tube smooth muscle and ciliary beat frequency in the airway epithelial cells via cGMP pathway (17, 18, 26). Also, another study showed that eNOS is expressed in all of ciliated tissues of rat (27). So, consider to these data, researchers suggest that NO may control ciliary activity by NO-cGMP signaling pathway (26). The previous study hypothesized that high concentrations of NO in fallopian tube may have cytotoxic effects on cells that consequently lead to deleterious alterations of tubal tissue and development of ectopic pregnancy (56, 57).

In the present study, high levels of luminal eNOS protein expression in ectopic pregnancy group support the importance of this enzyme for transport of the gametes and blastocyst within the fallopian tube and it’s over expression as a possible cause of ectopic implantation in these patients. The present study did not show any differences in endothelial micro vascular of eNOS concentrations in fallopian tubes tissue between all groups, suggesting that existence of eNOS in endothelial wall of blood vessels is not a essential factor in the pathogenesis of ectopic pregnancy. 

In this study we did not observe the expression of eNOS in smooth muscle cells of fallopian tube. Hence, we can suggest that NO produced by eNOS in luminal epithelium is very important in ciliary beat frequency and consequently over expression of eNOS may affect the transport of gametes or blastocyst within the fallopian tube and may lead to ectopic pregnancy. These results do not deny the paracrine effect of NO produced by luminal epithelial cells and endothelial lining of the vascular bed that affect the underlying smooth muscle layer. This finding is consistent with previous studies (58). So contraction decrease may lead to decrease the movement of blastocyst and ectopic implantation.

Though, eNOS expression in moderate amounts is essential for ciliary beating and smooth muscle contraction, but over expression of eNOS and consequently high concentration of NO in the fallopian tubes of patients with ectopic pregnancy may have cytotoxic effects on tissue and could change the ciliary activity and smooth muscle cell contraction, which could result in maintaining of the gamete or blastocyst within the fallopian tube and development of EP.

In summary, this is the first study that demonstrated the high levels of eNOS expression in FTs of ectopic pregnancy group compared with non-pregnant women at luteal phase of menstrual cycle and healthy pregnant groups. Based on our findings, it could be argued that basal generation of NO by epithelial cells may contribute significantly to the contraction and relaxation of the fallopian tube and hence actively participate in the process of gamete or blastocyst transport. Moreover, impairment of eNOS expression due to pathological factors may decrease ciliary activity and reduce contraction of smooth muscle of the fallopian tube and consequently affect the transport blastocyst through the tube which may lead to ectopic pregnancy.
